# MiR-330-5p inhibits intervertebral disk degeneration via targeting CILP

**DOI:** 10.1186/s13018-021-02582-4

**Published:** 2021-07-07

**Authors:** Shangzhi Li, Jinwei Liu, Liang Chen

**Affiliations:** 1 Department of Orthopaedics, Tianjin Hospital, Tianjin, 300211 People’s Republic of China; 2 Department of Orthopaedic Surgery, Liaocheng People’s Hospital, Liaocheng, 252000 Shandong People’s Republic of China

**Keywords:** miR-330-5p, CILP, Intervertebral disk degeneration

## Abstract

**Background:**

Intervertebral disk degeneration (IDD) is caused by nucleus pulposus (NP) degeneration and extracellular matrix (ECM) remodeling and cartilage intermediate layer protein (CILP) expression has been confirmed to be increased in IDD. This study is mainly conducted to clarify the mechanism of CILP in the NP cell degeneration and ECM remodeling in IDD.

**Methods:**

CILP expression in the degenerated NP tissues and cells is quantified by quantitative real-time PCR and western blot. CILP function is assessed by cell cycle assay, 3-(4,5-Dimethylthiazol-2-yl)-2,5-diphenyltetrazolium bromide assay and flow cytometry, β-galactosidase staining, and the detection of ECM-related molecules aggrecan, collagen type I, collagen type II, MMP-3, and MMP-9 expression is accomplished by qRT-PCR. The potential mechanism is authenticated by dual-luciferase reporter gene assay.

**Results:**

CILP was increased in the degenerated NP tissues and cells, and the knockdown of CILP promoted the NP cell cycle, increased cell activity, and repressed cell apoptosis and repressed cell senescence and ECM production. Moreover, miR-330-5p targeted the CILP 3′-untranslated region, and miR-330-5p negatively regulated CILP expression. Moreover, the overexpression of miR-330-5p repressed NP cell degeneration and ECM remodeling to relieve IDD by downregulating CILP.

**Conclusion:**

MiR-330-5p represses NP cell degeneration and ECM remodeling to ameliorate IDD by downregulating CILP.

## Introduction

Low back pain is a worldwide challenge that poses a serious threat to the physical and mental health of humans and imposes a burden on the social economy [1, 2]. Low back pain is mainly caused by intervertebral disk degeneration (IDD) [3]. IDD is caused by multiple factors, among which the degeneration of the nucleus pulposus (NP) and the remodeling of extracellular matrix (ECM) are the main factors for the acceleration of the development of IDD [4, 5]. Thus, the in-depth exploration of the molecular mechanism that caused NP degeneration and ECM remodeling was expected to alleviate IDD.

Cartilage intermediate layer protein (CILP) encodes cartilage intermediate layer protein and it has been corroborated that the abnormal expression of CILP is associated with a variety of human diseases [6, 7]. Recently, Ryan et al. have authenticated that the CILP gene polymorphism increases the risk of IDD through a meta-analysis [8]. Seki et al. have corroborated that the overexpression of CILP in NP promotes disk degeneration, implying that CILP is a direct factor in aggravating IDD [9]. Importantly, another research demonstrates that the abnormal expression of CILP in NP cells participates in the regulation of ECM synthesis in IDD [10]. Interestingly, our data also authenticated that CILP was upregulated in the degenerated NP tissues and cells, and we further proved that the knockdown of CILP repressed NP cell degeneration and ECM remodeling. However, the specific mechanism of CILP that regulated the NP cell degeneration and ECM remodeling in IDD remained unclear.

On the basis of the above findings, we in depth probed into the mechanism of CILP in regulating the NP cell degeneration and ECM remodeling in IDD, which might provide new potential biomarkers for the relief of IDD.

## Materials and methods

### Clinical samples

All the NP tissue samples were from the patients with degenerative disk disease who underwent discectomy after acquiring written informed consent from patients. This study was approved by the Ethics Committee of Liaocheng People’s Hospital.

### Cell culture

NP cells were purchased from Procell (Wuhan, China) and the cells were put in the Dulbecco’s modified Eagle medium (DMEM, ThermoFisher Scientific, WA, USA) with the addition of 10% fetal bovine serum (FBS, ThermoFisher Scientific, WA, USA) and 1% penicillin-streptomycin at 37 °C, 5% CO_2_.

### Quantitative real-time PCR

Based on the instructions of the reagent manufacturer, TRIzol (Invitrogen, Carlsbad, USA) reagent was applied to extract the total RNA from the NP tissues and cells. After the quality of RNA were analyzed by a NanoDrop ND1000 UV-VIS spectrophotometer (NanoDrop, Wilmington, USA), the high-quality RNAs were transcribed into cDNA using a TaqMan® MicroRNA Reverse Transcription Kit (ThermoFisher Scientific, WA, USA) or a reverse transcription cDNA kit (ThermoFisher Scientific, WA, USA). Subsequently, real-time PCR was conducted on the ABI 7300 Real-Time qPCR system (ABI company, New York, USA) using an SYBR Green PCR kit (ThermoFisher Scientific, WA, USA). U6 and β-actin were the internal references. The 2^−∆∆CT^ method was conducted to test the relative level of different molecules. All the primers sequences were exhibited in Table 1.

**Table 1:** The sequences of all primers used in qRT-PCR

Gene name	Primer sequence (5′-3′)
CILP	Forward: GCAAAAGCATCCTGAAGATCAC
	Reverse: GGAGTCTCTGCCCTCACAAAC
ACAN	Forward: ACCAGACTGTCAGATACCCC
	Reverse: CATAAAAGACCTCACCCTCC
COL1A1	Forward: TGACCTCAAGATGTGCCACT
	Reverse: ACCAGACATGCCTCTTGTCC3
COL2A1	Forward: ATTGCCTATCTGGACGAAGC
	Reverse: GCAGTGTACGTGAACCTGCT
MMP-3	Forward: GCATTGGCTGAGTGAAAGAGAC
	Reverse: ATGATGAACGATGGACAGATGA
MMP-9	Forward: GGGACGCAGACATCGTCATC
	Reverse: GGGACCACAACTCGTCATCG
miR-330-5p	Forward: TCTCTGGGCCTGTGTCTTAG
	Reverse: CAGTGCGTGTCGTGGAGT
β-actin	Forward: GTGGGGCGCCCCAGGCACCA
	Reverse: CTTCCTTAATGTCACGCACGATTTC
U6	Forward: GTGCTCGCTTCGGCAGCACATATAC
	Reverse: AAAAATATGGAACGCTTCACGAATTTG

### Western blot

The NP tissues and the differently treated NP cells were lysed with RIPA buffer (Sangon Biotech, Shanghai, China). Next, the proteins were quantified by a BCA Protein Assay Kit (Abcam, Cambridge, UK), and then the proteins with different molecular weights were separated by sodium dodecyl sulfonate-polyacrylamide gel electrophoresis (SDS-PAGE) and then transferred into polyvinylidene fluoride (Millipore, Massachusetts, USA) membranes. The above membranes were incubated with 5% skim milk for approximately 1 h and further incubated with specific primary antibodies, including anti-CILP (Abcam, 1: 1000 dilution, ab192881) and anti-β-actin (Abcam, 1: 1000 dilution, ab8227) at 4 °C overnight. After being washed about two times, the membranes were further incubated with the secondary antibody (Abcam, 1: 2000 dilution, ab205718) for about 1 h at room temperature. Ultimately, the enhanced chemiluminescence reagents (Millipore, Boston, Massachusetts, USA) and Image J (National Institutes of Health, Bethesda, USA) were applied to observe and analyze all the protein bands.

### Cell transfection and different treatments

The si-CILP, miR-330-5p mimic, and pcDNA-CILP were from GenePharma Company (Shanghai, China). Lipofectamine 2000 (Invitrogen, Carlsbad, CA, USA) was applied to cell transfection. Cell transfection details were as follows: NP cells (1 × 10^6^ cells/well) were put in 6-well plates and then cultured for nearly 24 h, and then the above synthetic si-CILP, miR-330-5p mimic, and pcDNA-CILP was transfected into NP cells via Lipofectamine 2000.

To probe into the functions of miR-330-5p and CILP in NP cells, the cells were mixed with 20 ng/ml TNF-α for approximately 24 h, and then were transfected with si-CILP, miR-330-5p, and/or pcDNA-CILP.

### Flow cytometry analysis

For the cell cycle, the NP cells were fixed overnight using 70% ethanol. Then, the cells were stained with the addition of propidium iodide (ThermoFisher Scientific, WA, USA). Ultimately, FC500 flow cytometry (Beckman, California, USA) was applied to assess the cell cycle of NP cells.

For the cell apoptosis, the NP cells were washed three times. Seventy percent ethanol was applied to fix these cells and the cell density was adjusted to 1 × 10^6^ cells. Next, 5 μl of Annexiv-FITC (Nanjing Genechem Co, Ltd., China) was added and incubated in darkness at 37 °C for approximately 30 min. Then, propidium iodide (PI) (Nanjing Genechem Co, Ltd., China) was added and continue to incubate in darkness for nearly 30 min. FC500 flow cytometry (Beckman, California, USA) was applied to test NP cell apoptosis.

### 3-(4,5-Dimethylthiazol-2-yl)-2,5-diphenyltetrazolium bromide (MTT) assay

MTT assay was applied to assess the viability of NP cells. Specifically, the NP cells (1 × 10^6^) with different treatments were put in 6-well plates and cultured. Twenty microliters of MTT solution was put in each well at 37 ° C for approximately 1 h. After that, 150 μl of DMSO was put in each well. The viability of NP cells was quantified at 0, 24, 48, and 72 h through an enzyme-linked immunodetection (ThermoFisher Scientific, WA, USA).

### β-galactosidase staining

Based on the standard procedure of the reagent manufacturer, Senescence Cells Histochemical Staining Kit (Sigma-Aldrich, WA, USA) was applied to analyze the senescence of NP cells. Specifically, NP cells (8 × 10^3^) were put in 6-well plates. Then, the medium was discarded and the cells were then fixed in the stain-fixative at room temperature for approximately 15 min, then washed three times with PBS, and incubated in the dyeing working solution at 37 °C and 5% CO_2_. A microscope (Olympus, Tokyo, Japan) was applied to obtain the images and the Image J software was applied to test the positive cells.

### Dual-luciferase reporter gene analysis

The potential binding sites of miR-330-5p and CILP were discovered by bioinformatics software (ENCORI). Furthermore, the dual-luciferase reporter gene was applied to authenticate this interaction. Briefly, a luciferase reporter vector that contained the potential miR-330-5p binding sites of CILP 3′UTR was constructed, and then the above recombinant vector and miR-330-5p mimic or NC mimic (negative control) were co-transfected into NP cells via Lipofectamine 2000 (Sigma-Aldrich, WA, USA). After approximately 48 h, the luciferase activity was measured by a dual-luciferase reporter gene assay system (Promega, Wisconsin, USA).

### Statistical analysis

All data were exhibited as mean ± standard deviation. Unpaired Student t test was applied to assess the differences of the two groups, and one-way ANOVA followed by Tukey’s post-test was applied to analyze the differences of more than two groups. *P* < 0.05 represented statistical significance.

## Results

### CILP is upregulated in the degenerated NP tissues and cells

**Fig. 1:**
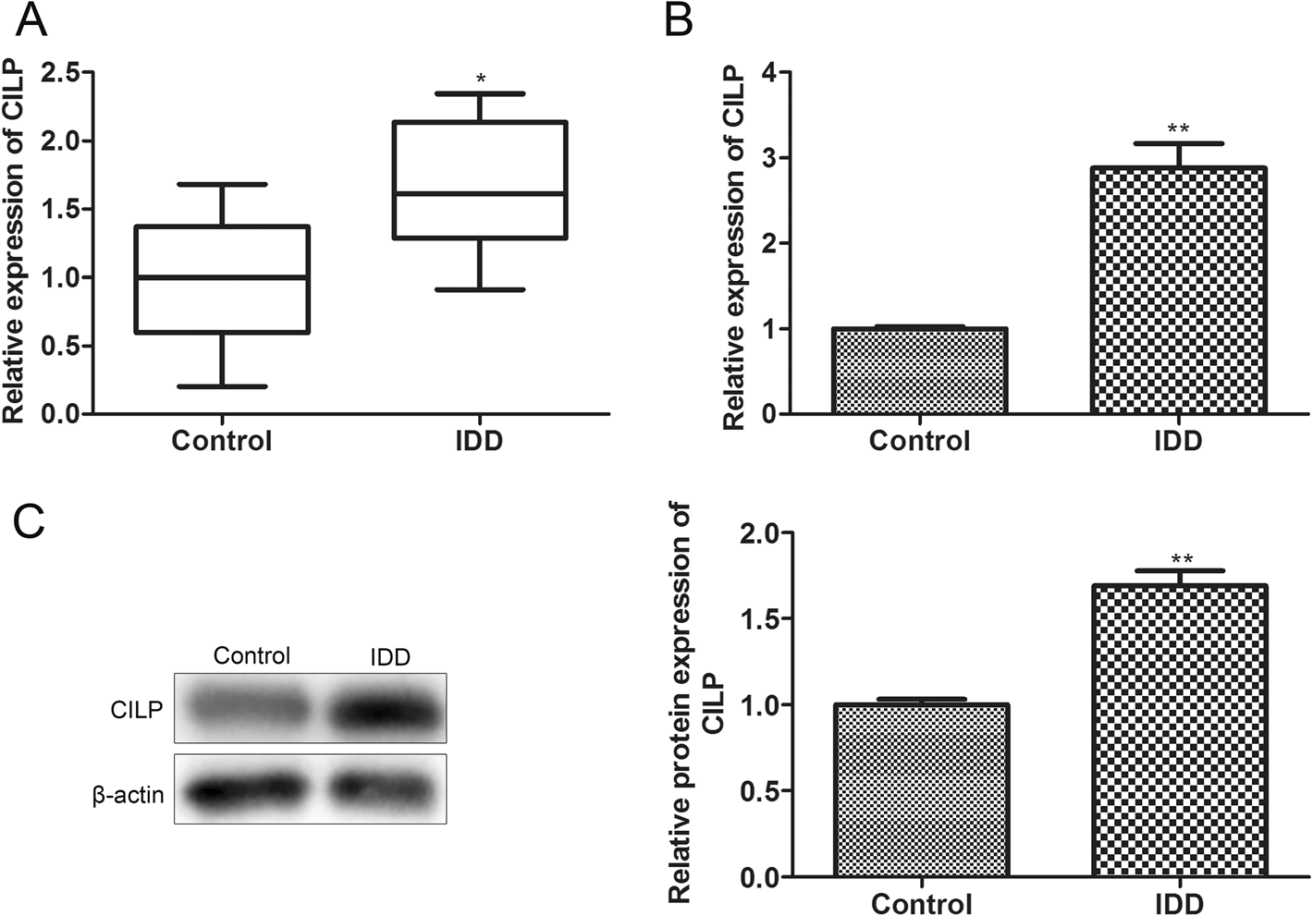
Expression of CILP in the degenerated nucleus pulposus (NP) tissues and cells. All the NP tissue samples and NP cells were harvested. (**A**-**B**) Quantitative real-time PCR (qRT-PCR) was applied to quantify the mRNA level of CILP in the degenerated NP tissues and cells. (**C**) Western blot was conducted to quantify the protein level of CILP in the degenerated NP tissues and cells. *P < 0.05, **P < 0.01 vs. control. NP, nucleus pulposus; IDD, intervertebral disk degeneration of three independent experiments

Although accumulating evidence authenticates that CILP has a regulatory function in various human diseases [11, 12], the function of CILP in the progression of IDD has not been fully elucidated. In the current research, we tested the mRNA level of CILP in the degenerated NP tissues and cells and the results corroborated that CILP was increased in the degenerated NP tissues and cells (Fig. 1A-B). Similarly, the analysis of western blot authenticated that CILP expression was elevated in the degenerated NP tissues and cells (Fig. 1C). Taken together, these findings corroborated that CILP was elevated in the degenerated NP tissues and cells.

### Knockdown of CILP promotes NP cell cycle, increases cell activity, and represses cell apoptosis

**Fig. 2:**
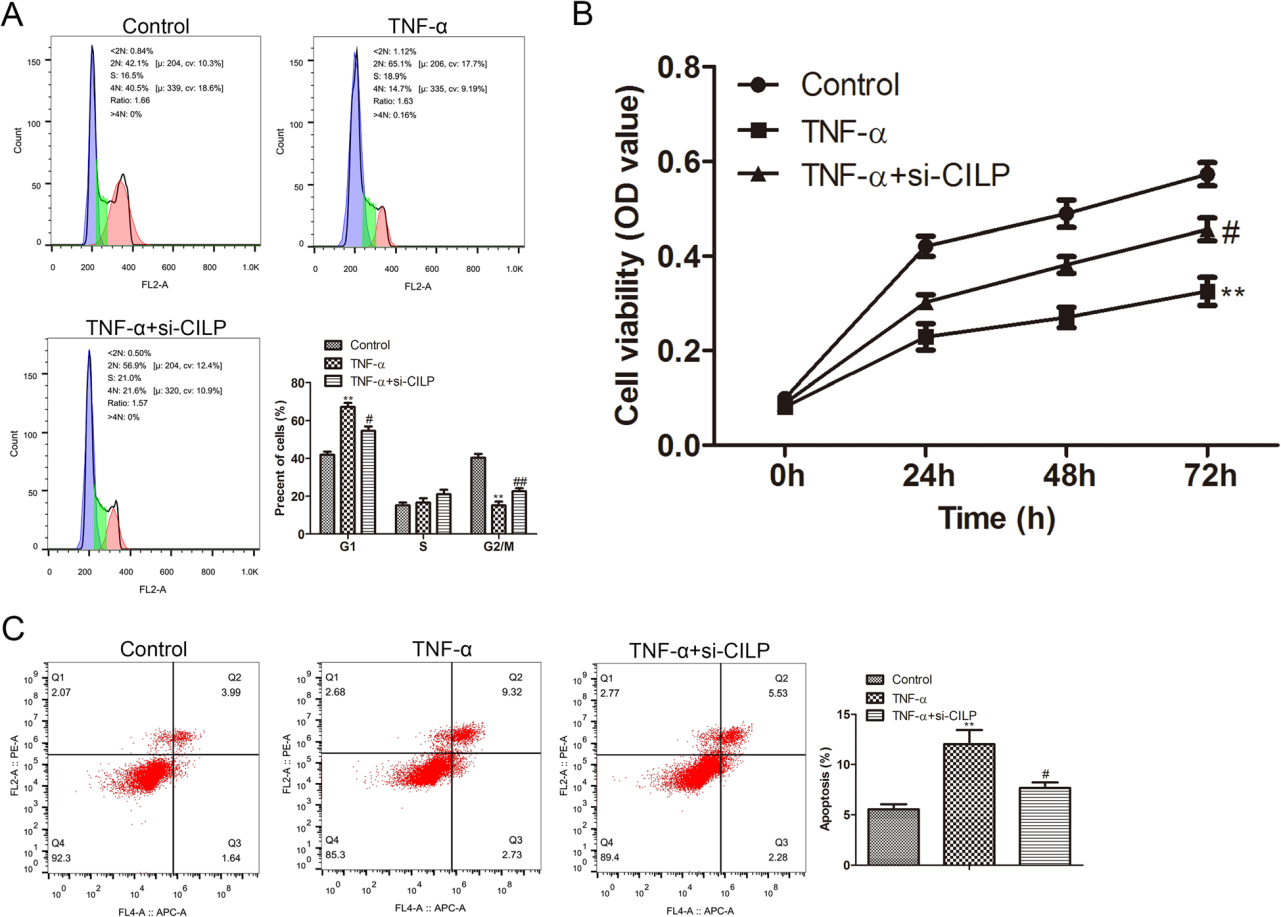
Influence of CILP in the cell cycle, activity, and apoptosis of NP cells. After the NP cells were mixed with 20 ng/ml TNF-α for approximately 24 h, the cells were transfected with si-CILP. (**A**) Flow cytometry was applied to test the cell cycle distribution of NP cells. (**B**) 3-(4,5-Dimethylthiazol-2-yl)-2,5-diphenyltetrazolium bromide (MTT) assay was applied to test NP cell activity at 0, 24, 48, and 72 h. (**C**) Flow cytometry was applied to analyze NP cell apoptosis. **P < 0.01 vs. control. ^#^*P* < 0.05, ^##^*P* < 0.01 vs. TNF-α of three independent experiments

To probe into the function of CILP in NP cell degeneration, si-CILP was transfected into the TNF-α-treated NP cells. As exhibited in Fig. 2A, cell cycle results revealed that the treatment of TNF-α led to cell cycle arrested in the G1 phase, and the transfection of si-CILP reversed this arrest. Meanwhile, the analysis of MTT corroborated that the treatment of TNF-α caused a decrease in NP cell activity, and the transfection of si-CILP reversed this decrease (Fig. 2B). Moreover, flow cytometry results authenticated that the treatment of TNF-α promoted NP cell apoptosis, and the transfection of si-CILP reversed this promotion (Fig. 2C). In conclusion, the above data corroborated that the knockdown of CILP promoted the NP cell cycle, increased cell activity, and repressed cell apoptosis.

### Knockdown of CILP represses the senescence and ECM production of NP cells

**Fig. 3:**
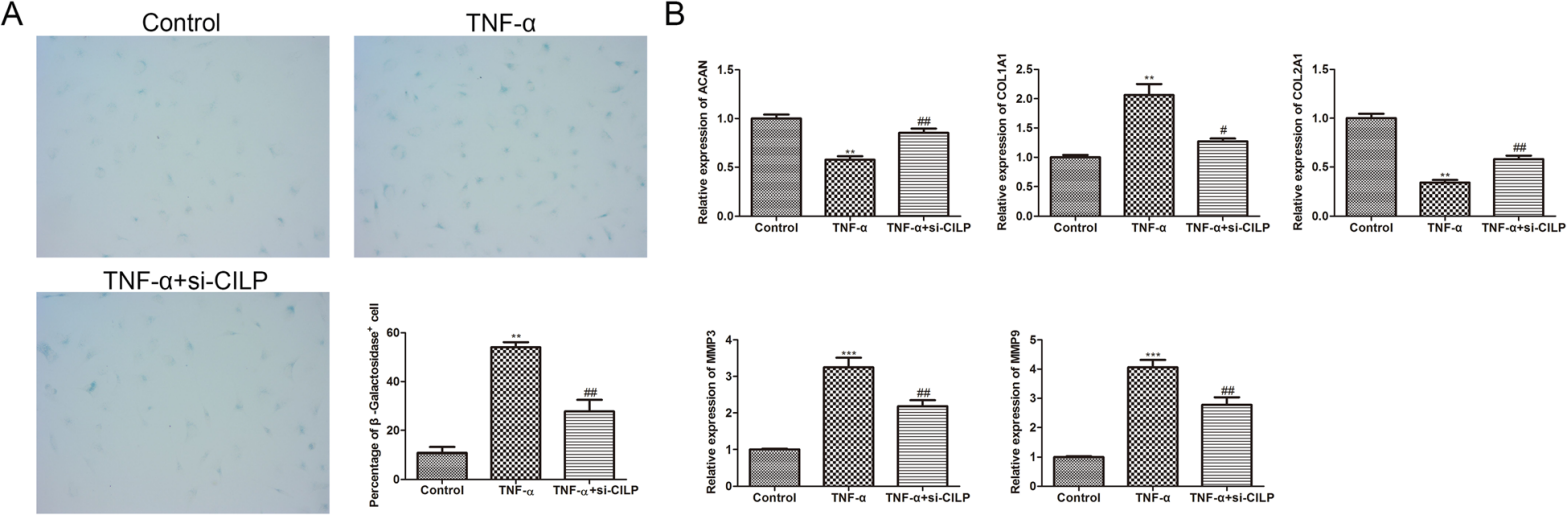
CILP affects the senescence and ECM production of NP cells. After the NP cells were mixed with 20 ng/ml TNF-α for approximately 24 h, the cells were transfected with si-CILP. (**A**) β-galactosidase staining was applied to assess NP cell senescence. (**B**) qRT-PCR was applied to quantify aggrecan (ACAN), collagen type I (COL1A1), collagen type II (COL2A1), MMP-3, and MMP-9 expressions in NP cells. **P < 0.01, ***P < 0.001 vs. control. ^#^*P* < 0.05, ^##^*P* < 0.01 vs. TNF-α of three independent experiments

Furthermore, the β-galactosidase staining corroborated that the treatment of TNF-α promoted the senescence of NP cells, and the transfection of si-CILP reversed this promotion (Fig. 3A). Previous studies have authenticated that aggrecan (ACAN), collagen type I (COL1A1), and collagen type II (COL2A1) are common markers used for the production of ECM [13, 14], and MMP-3 and MMP-9 have been reported to mediate the synthesis of ECM [15]. As exhibited in Fig. 3B, the treatment of TNF-α decreased the expressions of ACAN and COL2A1, and increased the expressions of COL1A1, MMP-3, and MMP-9, while these trends were reversed after the transfection of si-CILP. In general, the above experimental results corroborated that the knockdown of CILP repressed the senescence and ECM production of NP cells.

### MiR-330-5p binds to CILP

**Fig. 4:**
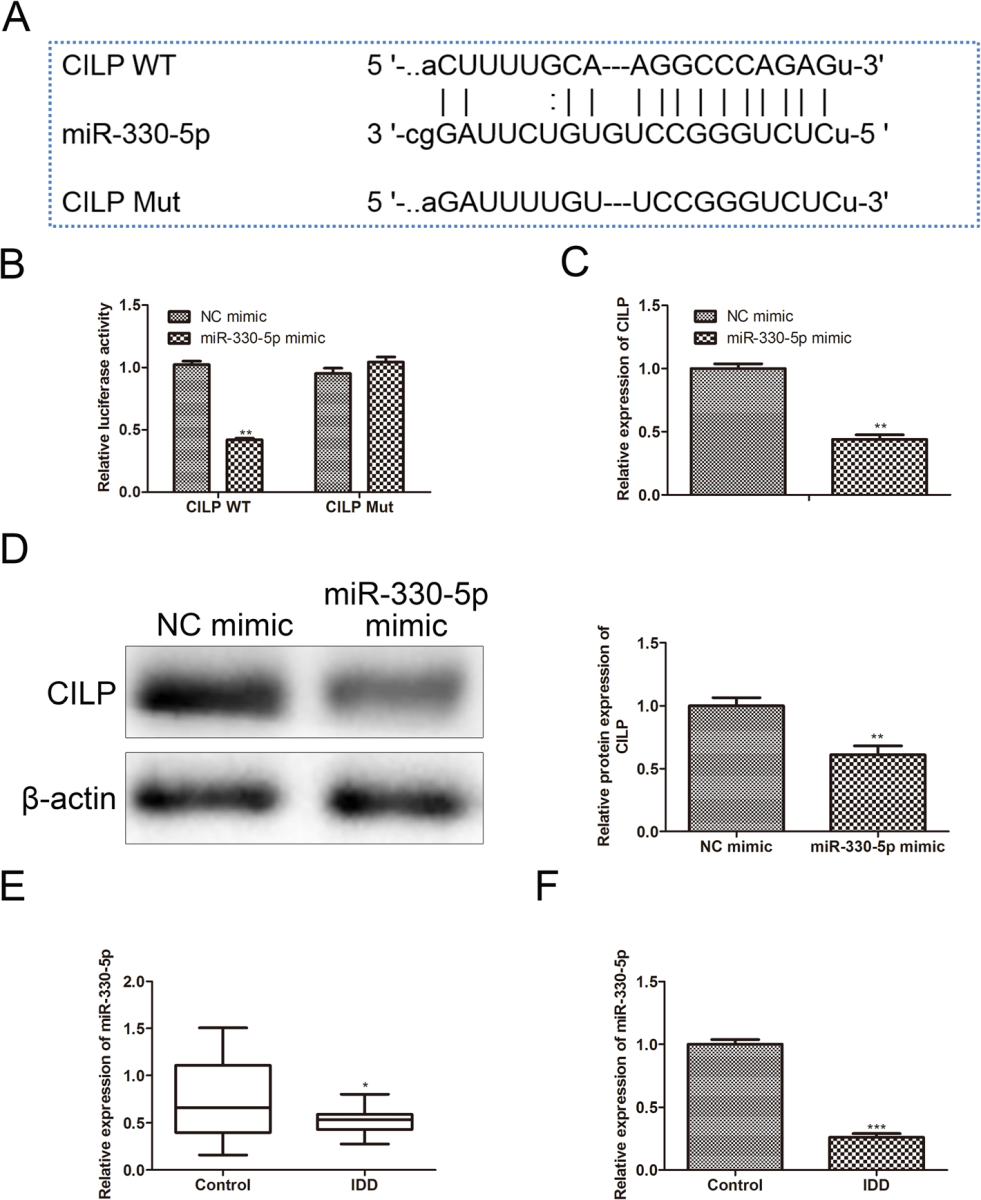
MiR-330-5p binds to CILP. (**A**) Bioinformatics software (ENCORI) predicted that hsa-miR-330-5p had the binding sites of the CILP 3′-untranslated region (UTR) region. (**B**) A dual-luciferase reporter gene analysis was conducted to test CILP relative luciferase activity. We transfected miR-330-5p mimic or NC mimic into NP cells. (**C**) qRT-PCR was conducted to quantify CILP expression. (**D**) Western blot was applied to quantify CILP protein level. (**E**-**F**) qRT-PCR was applied to measure miR-330-5p expressions in the degenerated NP tissues and cells. *P < 0.05 vs. control. **P < 0.01 vs. NC mimic. ***P < 0.001 vs. control. NC, negative control of three independent experiments

Next, we probed into the underlying mechanisms by which CILP played a regulatory function in NP cells. Bioinformatics software (ENCORI) predicted that hsa-miR-330-5p, hsa-miR-378g, and hsa-miR-514a-5p contained the binding sites of the CILP 3′UTR, and we selected hsa-miR-330-5p with the highest score for subsequent experiments, and the binding sites were exhibited in Fig. 4A. We further authenticated that the overexpression of miR-330-5p lessened the CILP WT relative luciferase activity, and had no remarkable changes in the CILP Mut relative luciferase activity (Fig. 4B). Similar to this finding, the overexpression of miR-330-5p lessened the CILP mRNA and protein levels (Fig. 4C-D). Moreover, we authenticated that miR-330-5p expression was decreased in the degenerated NP tissues and cells (Fig. 4E-F). Overall, these results corroborated that miR-330-5p bound to CILP and miR-330-5p negatively regulated CILP expression.

### MiR-330-5p/CILP axis regulates the cell cycle, activity, and apoptosis of NP cells

**Fig. 5:**
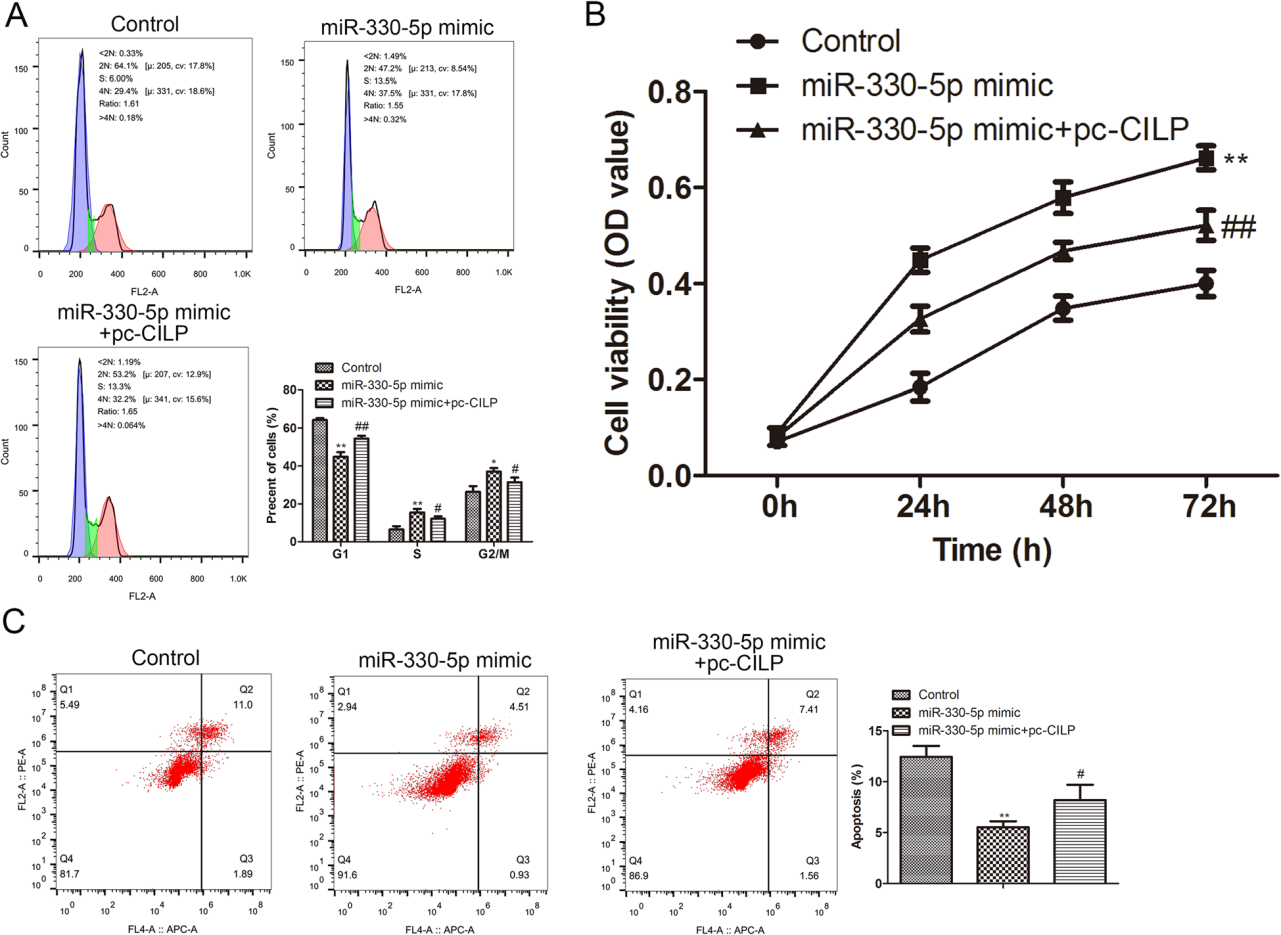
Influence of the miR-330-5p/CILP axis on the cell cycle, activity, and apoptosis of NP cells. MiR-330-5p mimic and/or pcDNA-CILP was transfected into NP cells. (**A**) Flow cytometry analysis was applied to test the cell cycle distribution of NP cells. (**B**) MTT was conducted to assess NP cell activity at 0, 24, 48, and 72 h. (**C**) Flow cytometry was applied to analyze NP cell apoptosis. *P < 0.05, **P < 0.01 vs. control. ^#^*P* < 0.05, ^##^*P* < 0.01 vs. miR-330-5p mimic. Pc-CILP, pcDNA-CILP of three independent experiments

Furthermore, we probed into how the miR-330-5p/CILP axis affected the development of IDD. MiR-330-5p mimic and/or pcDNA-CILP was transfected into the NP cells. As exhibited in Fig. 5A, the analysis of the cell cycle authenticated that the overexpression of miR-330-5p promoted the cell cycle, and the transfection of pcDNA-CILP reversed this trend. Similarly, MTT results corroborated that the overexpression of miR-330-5p increased the NP cell activity, and the transfection of pcDNA-CILP reversed this increase (Fig. 5B). Furthermore, the analysis of flow cytometry authenticated that the overexpression of miR-330-5p repressed the apoptosis of NP cells, and the transfection of pcDNA-CILP reversed this repression (Fig. 5C). Conclusively, the above data authenticated that the overexpression of miR-330-5p boosted the NP cell cycle, increased cell activity, and repressed cell apoptosis by downregulating CILP.

### MiR-330-5p/CILP axis regulates the senescence and ECM production of NP cells

**Fig. 6:**
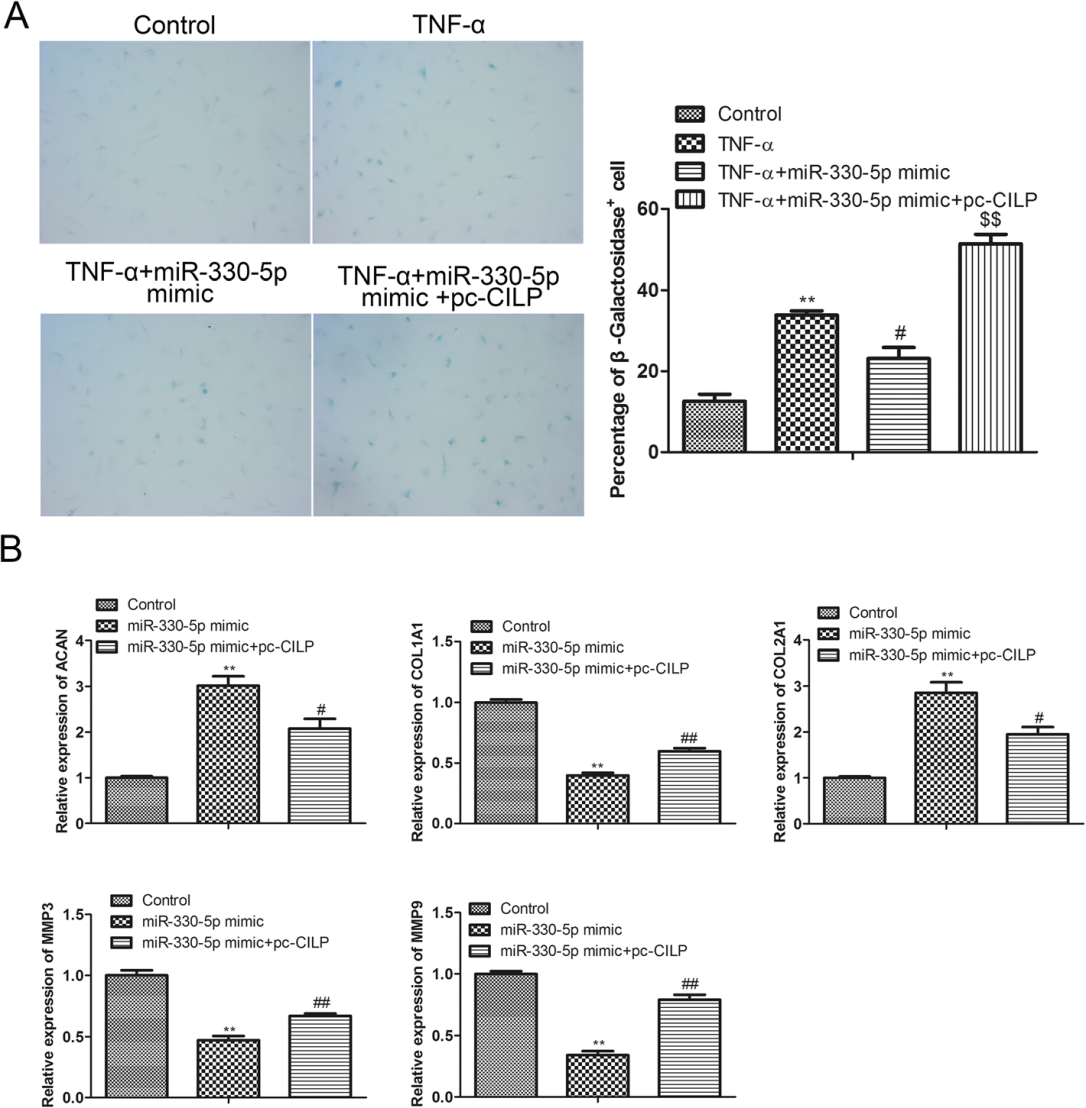
Influence of CILP on the senescence and ECM production of NP cells. MiR-330-5p mimic and/or pcDNA-CILP was transfected into the TNF-α-treated NP cells. (**A**) β-galactosidase staining was applied to analyze NP cell senescence. MiR-330-5p mimic and/or pcDNA-CILP was transfected into NP cells. (**B**) qRT-PCR was applied to quantify ACAN, COL1A1, COL2A1, MMP-3, and MMP-9 expressions in NP cells. **P < 0.01 vs. control. ^#^*P* < 0.05 vs. TNF-α or miR-330-5p mimic. ^##^*P* < 0.01 vs. miR-330-5p mimic. ^$$^*P* < 0.01 vs. TNF-α + miR-330-5p mimic of three independent experiments

Furthermore, as exhibited in Fig. 6A, the β-galactosidase staining results authenticated that the overexpression of miR-330-5p repressed the senescence of NP cells, while this repression was reversed after the transfection of pcDNA-CILP. Meanwhile, we measured the expressions of ACAN, COL1A1, COL2A1, MMP-3, and MMP-9 in NP cells and corroborated that the overexpression of miR-330-5p increased the expressions of ACAN and COL2A1, and decreased the expressions of COL1A1, MMP-3, and MMP-9, while these trends were reversed after the transfection of pcDNA-CILP (Fig. 6B). In general, these findings corroborated that the overexpression of miR-330-5p repressed the senescence and ECM production of NP cells by downregulating CILP.

## Discussion

IDD causes severe health and socioeconomic burdens and is still short of relieve strategies [16]. Thus, it is a need to find new therapeutic targets or directions to alleviate IDD. In the present research, we corroborated that CILP was elevated in the degenerated NP tissues and cells, and the knockdown of CILP repressed NP cell degeneration and ECM remodeling. For the mechanism exploration, our further study authenticated that the overexpression of miR-330-5p repressed NP cell degeneration and ECM remodeling by downregulating CILP. Our experimental results provided a new regulatory axis for IDD: miR-330-5p/CILP.

Accumulating evidence has corroborated that CILP is associated with the occurrence and development of IDD [10, 17]. Similar to these findings, our data authenticated that CILP expression was upregulated in the degenerated NP tissues and cells, and the knockdown of CILP repressed NP cell degeneration and ECM remodeling, implying that CILP was a critical regulator of IDD. However, the molecular mechanism by which CILP plays a function in IDD was not fully understood.

MicroRNAs (miRNAs) affect the expressions of target mRNAs via binding to their target mRNAs [18]. Previous studies have shown that miRNAs play regulatory roles in musculoskeletal disorders, including osteoarthritis and tendon injuries [19, 20]. Recently, increasing studies demonstrate that the abnormal expression of miRNAs is related to the development of IDD. For instance, miR-640 induces the degeneration of NP cells by targeting LRP1, thus promoting the development of IDD [21]. The upregulated miR-24-3p promotes the apoptosis of NP cells by binding IGFBP5 protein, ultimately accelerating the IDD process [22]. MiR-132 aggravates IDD by targeting GDF5 protein to promote ECM degradation in NP cells [23]. MiR-330-5p, as a common miRNA, has been found to regulate a variety of human diseases by binding to different proteins [24, 25]. In this study, we authenticated that miR-330-5p contained the binding sites of the CILP 3′UTR and miR-330-5p negatively regulated the CILP relative luciferase activity, implying that CILP was a downstream target molecule of miR-330-5p. On the basis of these findings, we further corroborated that the miR-330-5p/CILP axis regulated the NP cell degeneration and ECM remodeling through rescue experiments.

In summary, our data authenticated that CILP was elevated in the degenerated NP tissues and cells. Furthermore, the overexpression of miR-330-5p repressed NP cell degeneration and ECM remodeling by downregulating CILP. This study might provide new strategies and directions for the relief of IDD.

## Data Availability

All data generated or analyzed during this study are included in this published article.
